# A novel Rice QTL *qOPW11* Associated with Panicle Weight Affects Panicle and Plant Architecture

**DOI:** 10.1186/s12284-018-0246-x

**Published:** 2018-09-17

**Authors:** Satoshi Okada, Megumi Sasaki, Masanori Yamasaki

**Affiliations:** Food Resources Education and Research Center, Graduate School of Agricultural Science, Kobe University, Kasai, Hyogo 675-2103 Japan

**Keywords:** Rice yield, QTL, Panicle weight, Panicle branch, Panicle number

## Abstract

**Background:**

The improvement of rice yield is a crucial global issue, but evaluating yield requires substantial efforts. Rice yield comprises the following indices: panicle number (PN), grain number per panicle (GN), 1000-grain weight, and percentage of ripened grain. To simplify measurements, we analyzed one panicle weight (OPW) as a simplified yield index that integrates GN, grain weight, and percentage of ripened grain, and verified its suitability as a proxy for GN and grain weight in particular.

**Results:**

Quantitative trait locus (QTL) analysis using 190 recombinant inbred lines derived from Koshihikari (large panicle and small grain) and Yamadanishiki (small panicle and large grain), *japonica* cultivars detected three QTLs on chromosomes 5 (*qOPW5*), 7 (*qOPW7*) and 11 (*qOPW11*). Of these, *qOPW5* and *qOPW11* were detected over two years. *qOPW5* and *qOPW7* increased OPW, and *qOPW11* decreased it at Yamadanishiki alleles. A chromosome segment substitution line (CSSL) with a genomic segment from Yamadanishiki substituted in the Koshihikari genetic background harboring *qOPW5* increased grain weight. *qOPW11* had the largest genetic effect of QTLs, which was validated using a CSSL. Substitution mapping using four CSSLs revealed that *qOPW11* was located in the range of 1.46 Mb on chromosome 11. The CSSL harboring *qOPW11* decreased primary and secondary branch numbers, culm length, and panicle length, and increased PN.

**Conclusions:**

In this study, three QTLs associated with OPW were detected. The CSSL with the novel and largest QTL, *qOPW11*, differed in some traits associated with both panicle and plant architecture, indicating different functions for the meristem in the vegetative versus the reproductive stages. *qOPW5* coincided with an identified QTL for grain width and grain weight, suggesting that *qOPW5* was affected by rice grain size. OPW can be considered a useful trait for efficient detection of QTLs associated with rice yield.

**Electronic supplementary material:**

The online version of this article (10.1186/s12284-018-0246-x) contains supplementary material, which is available to authorized users.

## Background

Rice is one of the most important crops in the world because it is the staple food for half the global human population, especially in Asia. Rice yield has improved with breeding programs commencing in the 1960s with the utilization of the *semi-dwarf1* (Sasaki et al. 2002). Rice grain yield is a complex trait that is associated with many component traits, and the architecture of plant and panicle features such as tiller and panicle branch are important factors. Rice grain yield comprises four indices: panicle number (PN), grain number per panicle (GN), 1000-grain weight, and percentage of ripened grain (Xing and Zhang 2010; Ikeda et al. 2013), where PN is associated with the number of tillers and GN with panicle branching. It is crucial for next generation breeding programs to elucidate the quantitative trait loci (QTLs) and genes that determine plant and panicle architecture. To reduce the effort of separately examining the above four indices, we propose the use of a single combined index value, one panicle weight (OPW). As a simplified foothold-index for rice yield, it is roughly equivalent to the product of GN, grain weight, and the percentage of ripened grain; it also has a direct effect on yield per panicle. Use of this value can potentially contribute to efficient selection for high yield and may prove useful in genetic analyses.

The rice panicle has various characteristics such as primary and secondary branches, rachis, and spikelets (Crowell et al. 2016). Panicle architecture mainly consists of primary and secondary branches, and these traits are strongly related to GN (Ikeda et al. 2013; Peng et al. 2014; Rebolledo et al. 2016). Genes associated with the primary branch number (PBN) and the secondary branch number (SBN) have been cloned from natural and mutant rice variations. Examples of these are *Gn1a* (Ashikari et al. 2005), *DEP1* (Huang et al. 2009), *LAX1* (Komatsu et al. 2001), *OsSPL14* (Jiao et al. 2010; Miura et al. 2010), *SP1* (Li et al. 2009), and *APO1* (Ikeda et al. 2007; Ikeda-Kawakatsu et al. 2009). In addition, there exist many fine-mapped QTLs for panicle structure (Xing et al. 2008; Shan et al. 2009; Peng et al. 2014; Zhang et al. 2015; Sasaki et al. 2017). Genome-wide association studies for panicle architecture have recently been performed (Crowell et al. 2016; Rebolledo et al. 2016), and identification of genes associated with diversity of rice panicle has advanced. Of the QTLs and cloned genes associated with panicle architecture, some also affect tiller formation. Mutants of *MOC1* and *LAX1* reduce the number of both tillers and panicle branches (Komatsu et al. 2001; Li et al. 2003). In contrast, the near isogenic line (NIL) containing the *OsSPL14* gene was found to promote panicle branching and decrease tiller numbers (Jiao et al. 2010). Mutants of *SP1* and *OsARG* only affected panicle morphology (Li et al. 2009; Ma et al. 2013). The formation of tillers and panicle branches depends on common, independent, or interactive pathways, and it is likely that the genetic mechanisms controlling these are complex. Elucidation of these pathways is therefore useful for developing an optimized rice plant morphology. As the first step in the screening process, OPW is a useful and efficient measure to examine panicle architecture.

First, we evaluated OPW as a quantitative trait of rice grain yield and performed QTL analysis using recombinant inbred lines (RILs) derived from a cross between Koshihikari (large panicle and small grain) and Yamadanishiki (small panicle and large grain). As the unknown genetic factor for smaller panicle was of particular interest, these two cultivars were considered an appropriate pair for analysis purposes. Next, the major QTL detected on chromosome 11 was validated using chromosome segment substitution lines (CSSLs) to investigate plant and panicle architecture, and grain weight of CSSLs was measured. In the present study, our objectives were (1) to verify the use of OPW in evaluating some indices of rice grain yield – in particular, GN and grain weight; and (2) to elucidate the genetic factors for panicle development in Yamadanishiki.

## Results

### QTL Analysis

**Fig. 1:**
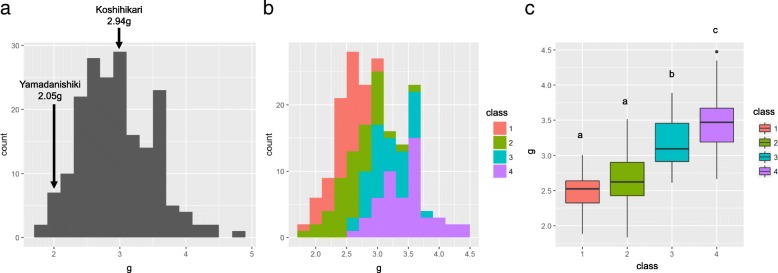
Histograms (**a** and **b**) and boxplot (**c**) for one panicle weight (OPW) of the recombinant inbred lines (RILs) from Koshihikari and Yamadanishiki in 2014. The histogram (**a**) was drawn using all 190 RILs, and the histogram (**b**) and boxplot (**c**) used 180 RILs of homozygous genotypes at the nearest single nucleotide polymorphism markers of *qOPW5* and *qOPW11*. The 180 RILs were classified into four classes: class 1: *qOPW5*_K and *qOPW11*_Y (45 lines), class 2: *qOPW5*_Y and *qOPW11*_Y (44 lines), class 3: *qOPW5*_K and *qOPW11*_K (40 lines) and class 4: *qOPW5*_Y and *qOPW11*_K (51 lines)

The histograms of the recombinant inbred lines (RILs) for OPW showed a continuous distribution, with transgressive segregations observed in 2014 and 2015 (Fig. 1a, Additional file 1: Figure S1A). Three QTLs were detected on chromosomes 5 (*qOPW5*), 7 (*qOPW7*), and 11 (*qOPW11*; Table 1). The genetic effect of *qOPW11* expressed more than 50% of phenotypic variance, and was the largest additive effect among the three QTLs (Table 1). The QTLs, *qOPW5* and *qOPW11* were detected in both 2014 and 2015. The Yamadanishiki alleles at *qOPW5* and *qOPW7*, and the Koshihikari allele at *qOPW11* increased OPW. In addition, no significant correlations between OPW and days to heading (DTH) were observed (Additional file 1: Figure S2), and the QTLs for OPW did not correspond to QTLs for DTH reported previously (Okada et al. 2017; Okada et al. 2018).

**Table 1:** The QTLs for one panicle weight

Chr.	QTL	Peak position (cM)	Confidence interval^a^ (cM)	Interval marker^b^	LOD		AE^c^ (g)		PVE^d^(%)	
					2014	2015	2014	2015	2014	2015
5	*qOPW5*	128.31	124.3–129.3	aa05000868-aa05001022	3.32	5.19	−0.10	−0.12	3.4	5.5
7	*qOPW7*	78.31	68.1–88.5	aa07001934-aa07005234	3.70	2.36^e^	− 0.13	−0.08	5.6	2.9
11	*qOPW11*	88.81	88–90.3	aa11004506-aa11005083	37.67	36.13	0.42	0.36	55.1	51.2

^a^ Confidence intervals showed total length of 1-LOD support interval of detected QTLs in 2014 and 2015

^b^ Interval markers showed the markers on either side of confience intervals

^c^ Additive effect. In case of the positive AE, the trait value incresed in the Koshihikari allele

^d^ Phenotypic variance explained

^e^ No significant LOD

The RILs were divided into four groups by the homozygous genotypes of the nearest markers for *qOPW5* and *qOPW11* (Fig. 1b and c, Additional file 1: Figure S1B and C). The effect of *qOPW5* was clearly visible in the multiple comparison test with *qOPW11*_K (classes 3 and 4; Fig. 1c), but the epistatic interaction between *qOPW5* and *qOPW11* was not significant (*P* = 0.087) in the three-way ANOVA test (Table 2). Class 4 (*qOPW5*_Y and *qOPW11*_K) showed the largest OPW of the four groups (Fig. 1b and c). This tendency was consistent over two years (Fig. 1 and Additional file 1: Figure S1).

**Table 2:** The result of three-way ANOVA for OPW of RILs

	F value	*P*
*qOPW5*	54.21	< 0.001
*qOPW11*	357.02	< 0.001
Year	48.14	< 0.001
*qOPW5 × qOPW11*	2.94	0.087
*qOPW5 × Year*	0.00	0.983
*qOPW11* × Year	0.95	0.330
*qOPW5* × *qOPW11* × Year	0.42	0.519

### Validation of *qOPW11*

**Fig. 2:**
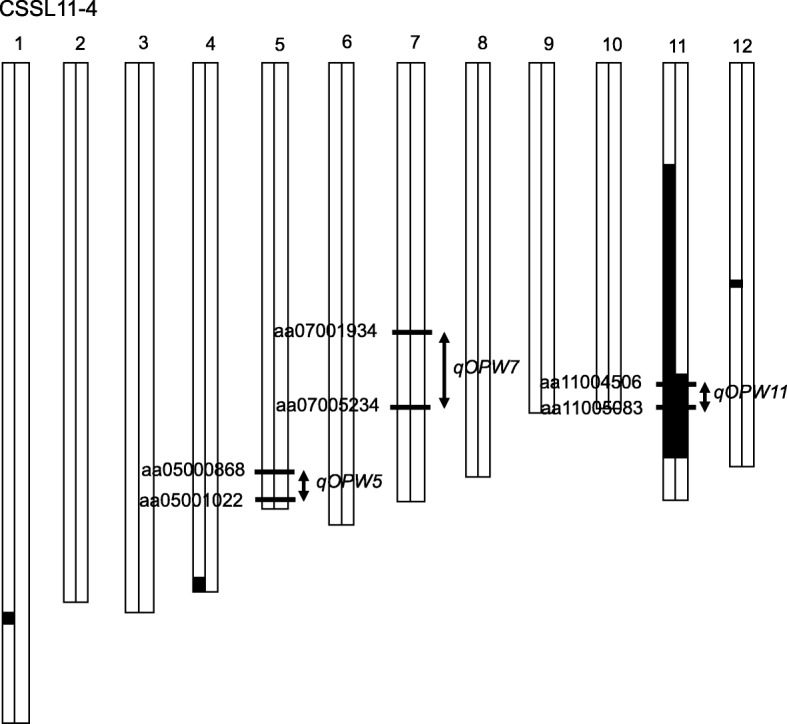
Graphical genotype of CSSL11–4 in 2015. Black and white blocks represent the genomic segments of Yamadanishiki and Koshihikari, respectively. The single nucleotide polymorphism markers show the interval markers around the detected QTLs for one panicle weight

**Fig. 3:**
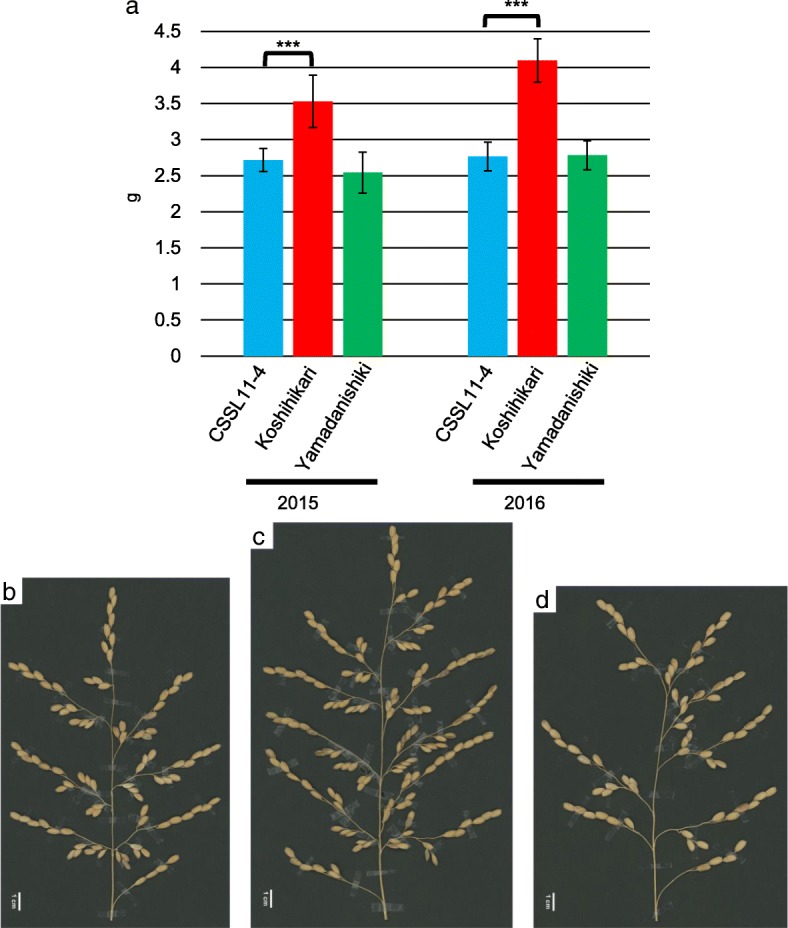
One panicle weights measured in CSSL11–4, Koshihikari, and Yamadanishiki (**a**) and panicles of CSSL11–4 (**b**), Koshihikari (**c**), and Yamadanishiki (**d**). *** represents a significant difference at *P* <  0.001

CSSL11–4 (the CSSL harboring the *qOPW11* region from Yamadanishiki in the Koshihikari genetic background) was selected to validate *qOPW11*, with the graphical genotypes represented in Fig. 2. The OPW of CSSL11–4 was significantly lower than that of Koshihikari in both 2015 and 2016 (Fig. 3a). The panicle of CSSL11–4 was smaller than that of Koshihikari but similar to that of Yamadanishiki (Fig. 3b, c, and d).

### Yield and Plant Traits of CSSLs Harboring *qOPW5* or *qOPW11*

First, we measured 100-grains weight (GWt) of CSSL5–5 (the CSSL harboring the *qOPW5* region from Yamadanishiki in the Koshihikari genetic background; Additional file 1: Figure S3), CSSL11–4 and parents. As the result, GWt of CSSL5–5 was heavier than that of Koshihikari over three years (Table 3). GWt of CSSL11–4 in 2015 was larger than that of Koshihikari in 2016 and 2017, and we also detected significant difference in 2016 and 2017; but differences between CSSL11–4 and Koshihikari were smaller (Table 3).

**Table 3:** One hundred grain weight of parents and CSSLs harboring qOPW5 and qOPW11

	100-grain weight^a^ (g)						
	CSSL5–5		CSSL11–4		Koshihikari		Yamadanishiki
2015^b^	2.39 ± 0.03***		2.38 ± 0.02***		2.20 ± 0.06		2.80 ± 0.05
2016^b^	2.25 ± 0.04***		2.23 ± 0.06**		2.16 ± 0.04		2.72 ± 0.05
2017	2.36 ± 0.02***		2.27 ± 0.03*		2.21 ± 0.03		2.81 ± 0.05

Astarisks represented significant difference compared with CSSLs and Koshihikari determined by Dannett’s test (“*”; *P* < 0.05, “**”; *P* < 0.01 and “***”; *P* < 0.001)

^a^ Phenotype data indicated “mean ± SD”

^b^ The data in 2015 and 2016 were cited by Okada et al. (2018)

**Fig. 4:**
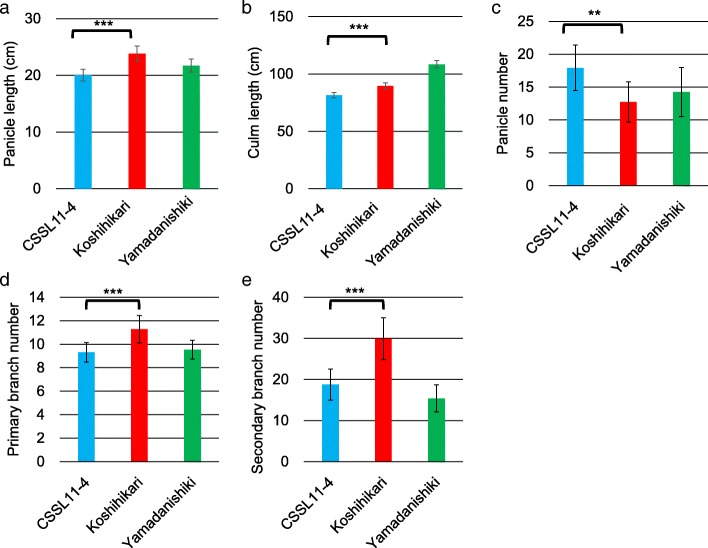
Pleiotropy of *qOPW11* with the panicle length (**a**), culm length (**b**), panicle number (**c**), primary branch number (**d**), and secondary branch number (**e**) of CSSL11–4, Koshihikari, and Yamadanishiki. Each trait was tested between CSSL11–4 and Koshihikari. ** represents *P* <  0.01 and *** represents *P* <  0.001

Next, we measured some traits associated with plant and panicle architecture for CSSL11–4. Panicle length (PL), PBN, SBN, and culm length (CL) in CSSL11–4 all were smaller than in Koshihikari, but PN in CSSL11–4 was greater (Fig. 4).

### Substitution Mapping of *qOPW11*

**Fig. 5:**
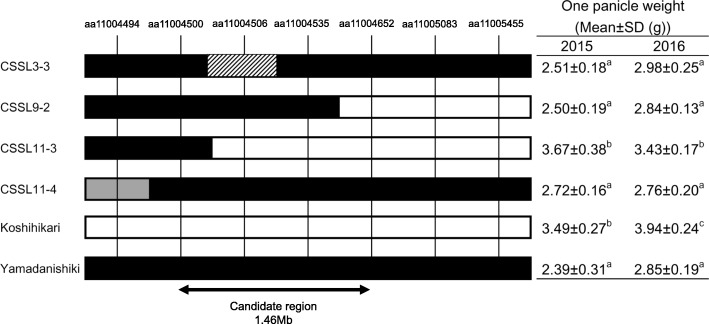
Substitution mapping of *qOPW11* using the chromosome segment substitution lines (CSSLs) on chromosome 11. Black, white, and gray blocks represent the Yamadanishiki homozygous genotypes, Koshihikari homozygous genotypes, and heterozygous genotypes, respectively. The striped block on CSSL3–3 shows the missing genotype at aa11004506. One panicle weight values are shown for 2015 and 2016. Different letters indicate significant differences between samples in the Tukey-Kramer test

Four CSSLs that had a genomic segment around *qOPW11* (Fig. 5) were used for substitution mapping of *qOPW11*. Of the four lines, the OPWs of three lines (CSSL3–3, 9–2, 11–4) were less than that of Koshihikari in both 2015 and 2016. In contrast, the OPWs of CSSL11–3 were greater than those of these three CSSLs in both years, similar to Koshihikari in 2015, and slightly less than Koshihikari in 2016. As a result, *qOPW11* was mapped between aa11004500 and aa11004652, with a physical distance of approximately 1.46 Mb.

## Discussion

Panicle size and architecture are important elements for rice yield. We evaluated one trait related to the panicle, OPW, and examined its suitability for efficient genetics analysis of rice yield. OPW incorporates the following traits: GN, grain size, percentage of ripened grain, and the branching frame of the panicle. The use of OPW facilitated the detection of responsible QTLs using RILs and narrowed down the number of CSSLs, allowing the efficient investigation of panicle and yield components. We detected three QTLs on chromosomes 5, 7, and 11, of which *qOPW5* and *qOPW11* had consistent effects on OPW (Table 1). No loci associated with panicle traits around *qOPW5* and *qOPW11* are on record in the Q-TARO (Yonemaru et al. 2010) or Gramene databases (http://www.gramene.org/), suggesting that the two QTLs are novel. Phenotypic variance of OPW in the RILs was mostly explained by *qOPW11*, and the Yamadanishiki allele at *qOPW11* had an effect of decreasing the OPW (Table 1). Therefore, it appears that *qOPW11* causes short panicles in Yamadanishiki. *qOPW11* was mapped at 1.46 Mb on chromosome 11 by substitution mapping with CSSLs (Fig. 5), and provided a foothold for the map-based cloning.

A QTL for grain width and grain weight was reported by Yoshida et al. (2002), Nagata et al. (2015), and Okada et al. (2017), but they did not identify a QTL associated with panicle traits around *qOPW5*. A QTL (*qGWh5*) for grain width was recently identified on chromosome 5 using the same CSSLs used in the present study (Okada et al. 2018). In the present study, it was elucidated that *qOPW5* clearly had an effect on grain weight by evaluating GWt over three years (Table 3). GWt of CSSL11–4 in 2015 was larger than GWt in other years (Table 3). Because *qGL11* of QTL had the largest effect on grain length in heterozygous region on chromosome 11 in CSSL11–4 in 2015 (Okada et al. 2017; Okada et al. 2018), it appears that *qGL11* of CSSL11–4 in 2015 affected GWt. However, CSSL11–4 had slightly heavier GWt than Koshihikari in 2016 and 2017. Therefore, *qOPW11* may also have an effect on grain weight, although the possibility exists that two tightly linked QTLs or Yamadanishiki segments in the genetic background influence each other.

The RILs harboring the Yamadanishiki allele at *qOPW5* and the Koshihikari allele at *qOPW11* showed the highest OPW among the genotype classes (Fig. 1b and c). When *qOPW11* contained the Koshihikari allele, the effect of *qOPW5* was clearly represented; however, the presence of an epistatic interaction between *qOPW5* and *qOPW11* was not clear (Fig. 1c, Table 2). It can therefore be concluded that *qOPW5* did not affect panicle architecture but did affect grain size and weight, resulting in the increase of panicle weight. The transgressive segregation of the histograms of OPW for the RILs was mostly explained by *qOPW5* and *qOPW11* (Fig. 1). Further analysis for yield of these QTLs with NILs and pyramiding lines is recommended, since genes associated with grain size also affect panicle traits (Hu et al. 2015; Si et al. 2016).

The phenotype for plant and panicle architecture of CSSL11–4 was clearly different from Koshihikari (Fig. 4). Because there is a positive correlation between PBN and GN (Peng et al. 2014; Rebolledo et al. 2016), we suggest that decrease of OPW in presence of the Yamadanishiki allele was caused by decrease of PBN. Therefore, *qOPW11* would affect panicle architecture. Moreover, it is clear that CSSL11–4 expressed a different plant architecture, with traits such as PN and CL derived from Koshihikari (Fig. 4). When the allele of *qOPW11* changes from the Yamadanishiki allele to the Koshihikari allele, PL and CL increase and PN decreases (Fig. 4). Yoshida et al. (2002) detected a major QTL for PN on chromosome 11 using double haploid lines derived from a cross between Reiho and Yamadanishiki. The QTL would correspond to *qOPW11* because of increasing PN at the Yamadanishiki allele. This suggests that *qOPW11* would have different functions for the meristem in the vegetative versus the reproductive stages, when the effect of *qOPW11*_K would suppress tiller and increase panicle branch. However, fine mapping for *qOPW11* should be conducted because it is possible that two tightly linked QTLs affect the measured traits.

Several *SPL* (*SQUAMOSA PROMOTER BINDING PROTEIN-LINE*) genes are associated with the formation of both panicle and tiller (Jiao et al. 2010; Miura et al. 2010; Wang et al. 2015; Si et al. 2016), and plant and panicle architecture are changed by the expression level of these genes (Wang et al. 2015; Wang and Zhang 2017). For example, with high expression levels of *OsSPL14* at both the vegetative and reproductive stages, tiller number decreased but PBN and SBN increased (Jiao et al. 2010; Miura et al. 2010). In addition, mutants of *D14*/*D88* and *D53* associated with strigolactone signaling also showed increased tiller numbers and decreased panicle size (Arite et al. 2009; Gao et al. 2009; Zhou et al. 2013). Therefore, *qOPW11* may also be involved with the pathways for both plant and panicle architecture.

## Conclusions

We detected three QTLs associated with rice yield using QTL analysis for the single trait OPW. Of the detected QTLs, *qOPW5* affected grain size and *qOPW11* affected panicle architecture. *qOPW11* caused the largest effect, clearly affecting both plant and panicle architecture, and was isolated to 1.46 Mb on chromosome 11. Further analysis of these QTLs will be beneficial in increasing rice yield and elucidating the development of rice plant morphology. OPW can be considered a useful trait for detection of QTLs associated with rice yield.

## Methods

### Plant Materials and Cultivation Conditions

We used two kinds of plant materials: (1) the recombinant inbred lines (RILs) derived from a cross between the Koshihikari (large panicle and small grain) and Yamadanishiki (small panicle and large grain) *japonica* cultivars, and (2) the chromosome segment substitution lines (CSSLs) from Yamadanishiki in the Koshihikari genetic background (Okada et al. 2017; Okada et al. 2018). All plants were cultivated in an experimental field located at the Food Resources Education and Research Center of Kobe University (Kasai City, Hyogo Prefecture, Japan) (34.880 N, 134.866E). The RIL population was cultivated in 2014 and 2015. A total of 190 RILs were genotyped with 312 DNA markers. Of these, 310 markers were identified by Okada et al. (2017) and an additional two markers were determined in the present study (C6_3000 and RM6704; Additional file 2: Tables S1 and S2).

The four CSSLs (CSSL3–3, CSSL9–2, CSSL11–3, and CSSL11–4) and the parent plants (Koshihikari and Yamadanishiki) were cultivated in 2015 and 2016 for validation and substitution-mapping of a major QTL for OPW on chromosome 11. Okada et al. (2018) identified genotypes of the four CSSLs.

### Phenotypic Evaluation

The OPW trait was measured for QTL analysis, validation, and substitution mapping of a QTL. Eight panicles were harvested per rice plant over 45 days after their flowering date, their weight was measured using an electronic balance to an accuracy of 0.01 g, and the average value of OPW was calculated. In the QTL analysis, this trait was evaluated for three plants per RIL. The data for days to heading (DTH) of RILs and CSSLs are shown in Additional file 1: Figure S2 and Additional file 2: Table S3. The QTLs for DTH of Koshihikari / Yamadanishiki crossed populations were previously identified for *qDTH3* on chromosome 3 and *qDTH6* on chromosome 6 (Okada et al. 2017; Okada et al. 2018). *qDTH3* at the Yamadanishiki allele had a very large genetic effect which increased at about 20 days, and expressed phenotypic variance of DTH in the RIL population beyond 75%. Histograms of RILs therefore show two peaks. The DTHs of used CSSLs showed a similar to that of Koshihikari because these lines had *qDTH3* homozygous at the Koshihikari allele.

Several traits were measured to evaluate the effect of *qOPW5* and *qOPW11*. First, GWt was measured in CSSL5–5, CSSL11–4 and parents in 2015, 2016 and 2017. The 2015 and 2016 values are published in Okada et al. (2018). CL, PL, PN, PBN, and SBN were measured on CSSL11–4 as well as the parent plants in order to examine the QTL effect on other agronomic traits of plant and panicle architecture.

### QTL Analysis and Statistical Analysis

QTL analysis was performed on 190 RILs using Windows QTL Cartographer 2.5 (Wang et al. 2012). QTLs were detected using the composite interval mapping method (Zeng 1994) with window size and walk speed set at 5 and 1 cM, respectively. The empirical threshold (α = 0.05) for logarithm of odds (LOD) values was determined from 1000 permutations (Churchill and Doerge 1994). Confidence intervals were calculated from a 1-LOD support interval.

For two of the detected QTLs (*qOPW5* and *qOPW11*), the 190 RILs were classified by the genotypes of the nearest markers (ac05000341 and aa11004535) on chromosomes 5 and 11 (Additional file 2: Table S1). The 180 homozygous lines were classified into four classes: class 1 – Koshihikari homozygous allele at *qOPW5* (*qOPW5*_K) and Yamadanishiki homozygous allele at *qOPW11* (*qOPW11*_Y) (45 lines); class 2 – Yamadanishiki homozygous allele at *qOPW5* (*qOPW5*_Y) and *qOPW11*_Y (44 lines); class 3 – *qOPW5*_K and Koshihikari homozygous allele at *qOPW11* (*qOPW11*_K) (40 lines); and class 4 – *qOPW5*_Y and *qOPW11*_K (51 lines). The 10 heterozygous RILs were excluded. Three-way ANOVA and multiple Tukey-Kramer comparisons were conducted with statistics software R 3.4.1 (R Core Team 2017) and histograms and boxplots were created using the R package ‘ggplot2’.

Comparison between the CSSLs harboring *qOPW5* or *qOPW11* and Koshihikari was performed by Dunnett’s test. Validation of *qOPW11* and evaluation of effects on other traits was carried out using a two-sided *t*-test for OPW, CL, PL, PN, PBN, and SBN between Koshihikari and CSSL11–4. Substitution mapping in the 2015 and 2016 results was statistically analyzed using one-way ANOVA.

## Additional files

Additional file 1: **Figure S1.** The histograms (A and B) and boxplot (C) for OPW of RILs from Koshihikari/Yamadanishiki in 2015. The histogram (B) and boxplot used 180 RILs homozygous at the nearest SNP markers of *qOPW5* and *qOPW11*. The 180 RILs were classified into four class; class1: *qOPW5*_K and *qOPW11*_Y (45 lines), class2: *qOPW5*_Y and *qOPW11*_Y (44 lines), class3: *qOPW5*_K and *qOPW11*_K (40 lines) and class 4: *qOPW5*_Y and *qOPW11*_K (51 lines). **Figure S2.** Scatter plots between OPW and days to heading for RILs in 2014 (A) and 2015 (A). No significant correlations were observed with Kendall’s rank correlation both years (2014; tau = 0.065, *P* = 0.19 and 2015; tau = − 0.076, *P* = 0.13). **Figure S3.** Graphical genotype of CSSL5–5. (PPTX 416 kb)

Additional file 2: **Table S1.** Genotypes of RILs. **Table S2.** Additional markers using genotypes of RILs. **Table S3** Days to heading of CSSLs and parents. (XLSX 212 kb)

## Abbreviations

ANOVA: Analysis of variance
*APO1*: *ABERRANT PANICLE ORGANIZATION 1*
CL: Culm length
CSSL: Chromosome segment substitution line
*D14*/*D88*: *DWARF 14*/*DWARF88*
*D53*: *DWARF 53*
*DEP1*: *DENSE AND ERECT PANICLE 1*
DTH: Days to heading
GN: Grain number per panicle
*Gn1a*: *GRAIN NUMBER 1a*
GWt: 100-grain weight
*LAX1*: *LAX PANICLE 1*
LOD: Logarithm of odds
*MOC1*: *MONOCULM 1*
NIL: Near isogenic line
OPW: One panicle weight
*OsARG*: *ARGINASE*
*OsSPL14*: *SOUAMOSA PROMOTER BINDING PROTEIN-LIKE 14*
PBN: Primary branch number
PL: Panicle length
PN: Panicle number
QTL: Quantitative trait locus
RIL: Recombinant inbred line
SBN: Secondary branch number
SNP: Single nucleotide polymorphism
*SP1*: *SHORT PANICLE 1*
*SPL*: *SQUAMOSA PROMOTER BINDING PROTEIN-LIKE*
