# Supplementary material for: A novel Rice QTL qOPW11 Associated with Panicle Weight Affects Panicle and Plant Architecture
Source: Rice (N Y). 2018 Sep 17;11:53. doi: 10.1186/s12284-018-0246-x (PMC6141410; doi:10.1186/s12284-018-0246-x)
Supplement: Supplementary file 1 — Figure S1. The histograms (A and B) and boxplot (C) for OPW of RILs from Koshihikari/Yamadanishiki in 2015. The histogram (B) and boxplot used 180 RILs homozygous at the nearest SNP markers of qOPW5 and qOPW11. The 180 RILs were classified into four class; class1: qOPW5_K and qOPW11_Y (45 lines), class2: qOPW5_Y and qOPW11_Y (44 lines), class3: qOPW5_K and qOPW11_K (40 lines) and class 4: qOPW5_Y and qOPW11_K (51 lines). Figure S2. Scatter plots between OPW and days to heading for RILs in 2014 (A) and 2015 (A). No significant correlations were observed with Kendall’s rank correlation both years (2014; tau = 0.065, P = 0.19 and 2015; tau = − 0.076, P = 0.13). Figure S3. Graphical genotype of CSSL5–5. (PPTX 416 kb) [file 12284_2018_246_MOESM1_ESM.pptx]

## Slide 1
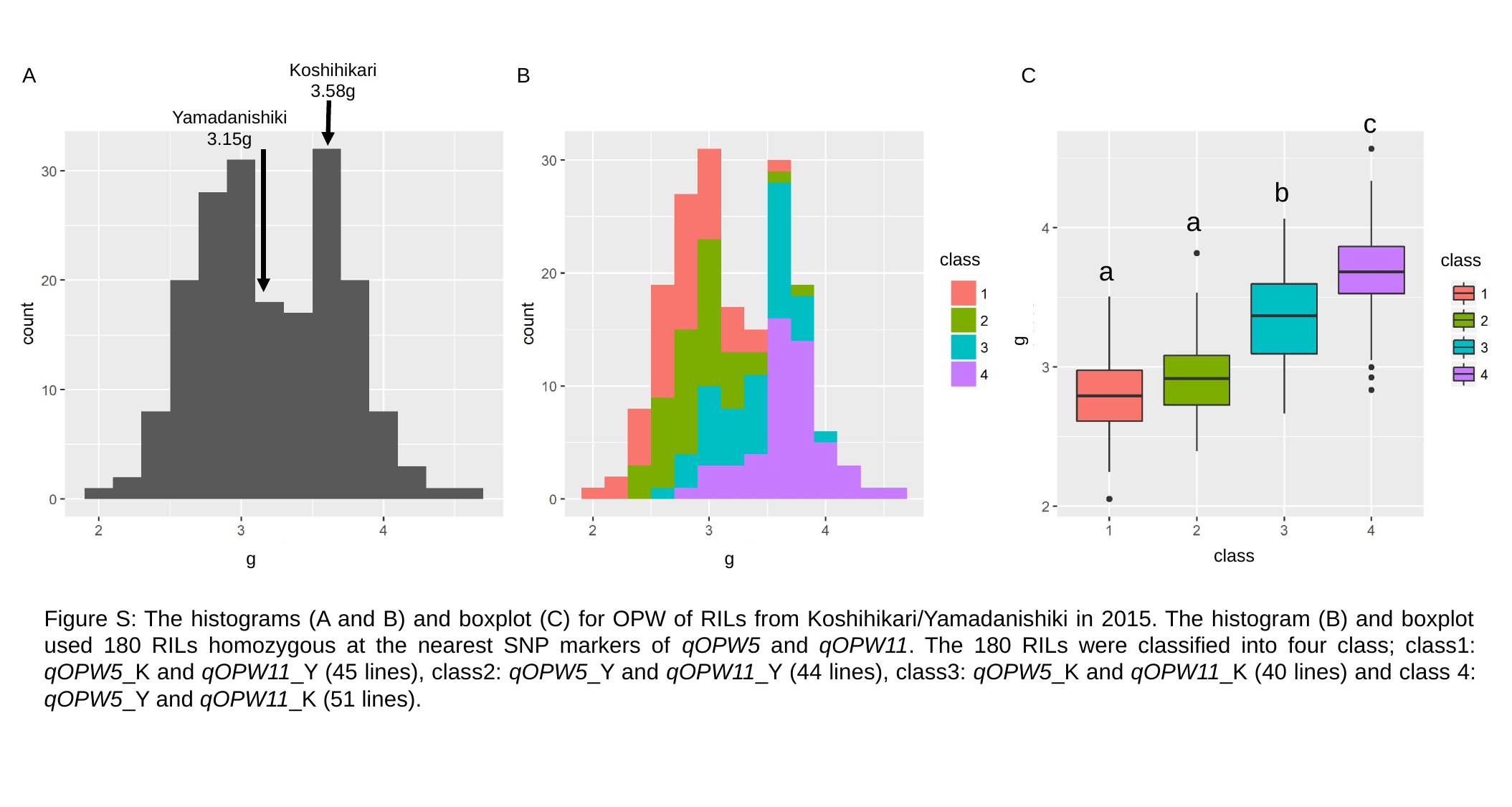

Koshihikari
3.58g
A
B
C
Yamadanishiki
3.15g
c
b
a
class
class
a
 g
 class
 g
 g
Figure S: The histograms (A and B) and boxplot (C) for OPW of RILs from Koshihikari/Yamadanishiki in 2015. The histogram (B) and boxplot used 180 RILs homozygous at the nearest SNP markers of qOPW5 and qOPW11. The 180 RILs were classified into four class; class1: qOPW5_K and qOPW11_Y (45 lines), class2: qOPW5_Y and qOPW11_Y (44 lines), class3: qOPW5_K and qOPW11_K (40 lines) and class 4: qOPW5_Y and qOPW11_K (51 lines).

## Slide 2
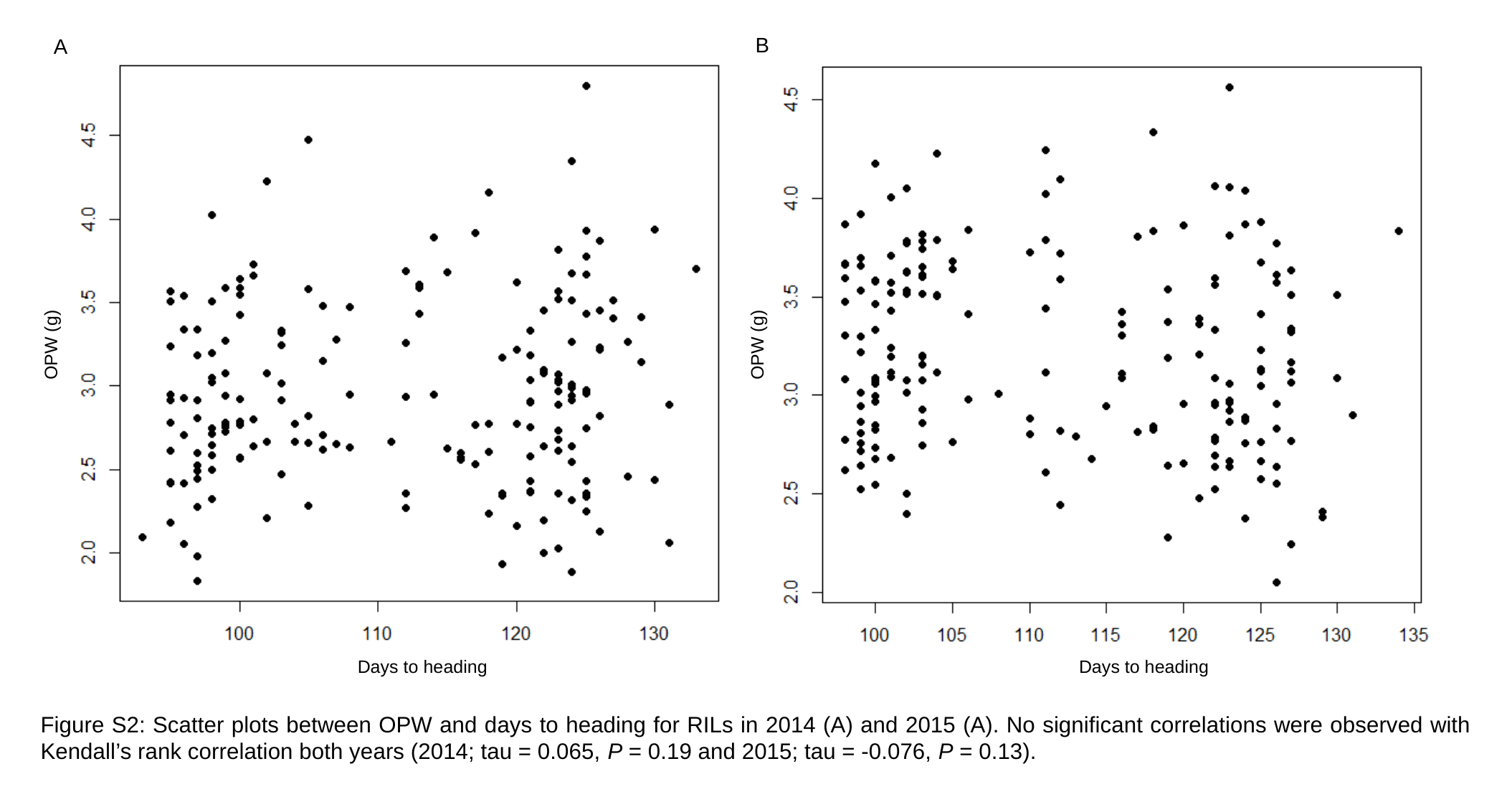

B
 A
 OPW (g)
 OPW (g)
 Days to heading
 Days to heading
Figure S2: Scatter plots between OPW and days to heading for RILs in 2014 (A) and 2015 (A). No significant correlations were observed with Kendall’s rank correlation both years (2014; tau = 0.065, P = 0.19 and 2015; tau = -0.076, P = 0.13).

## Slide 3
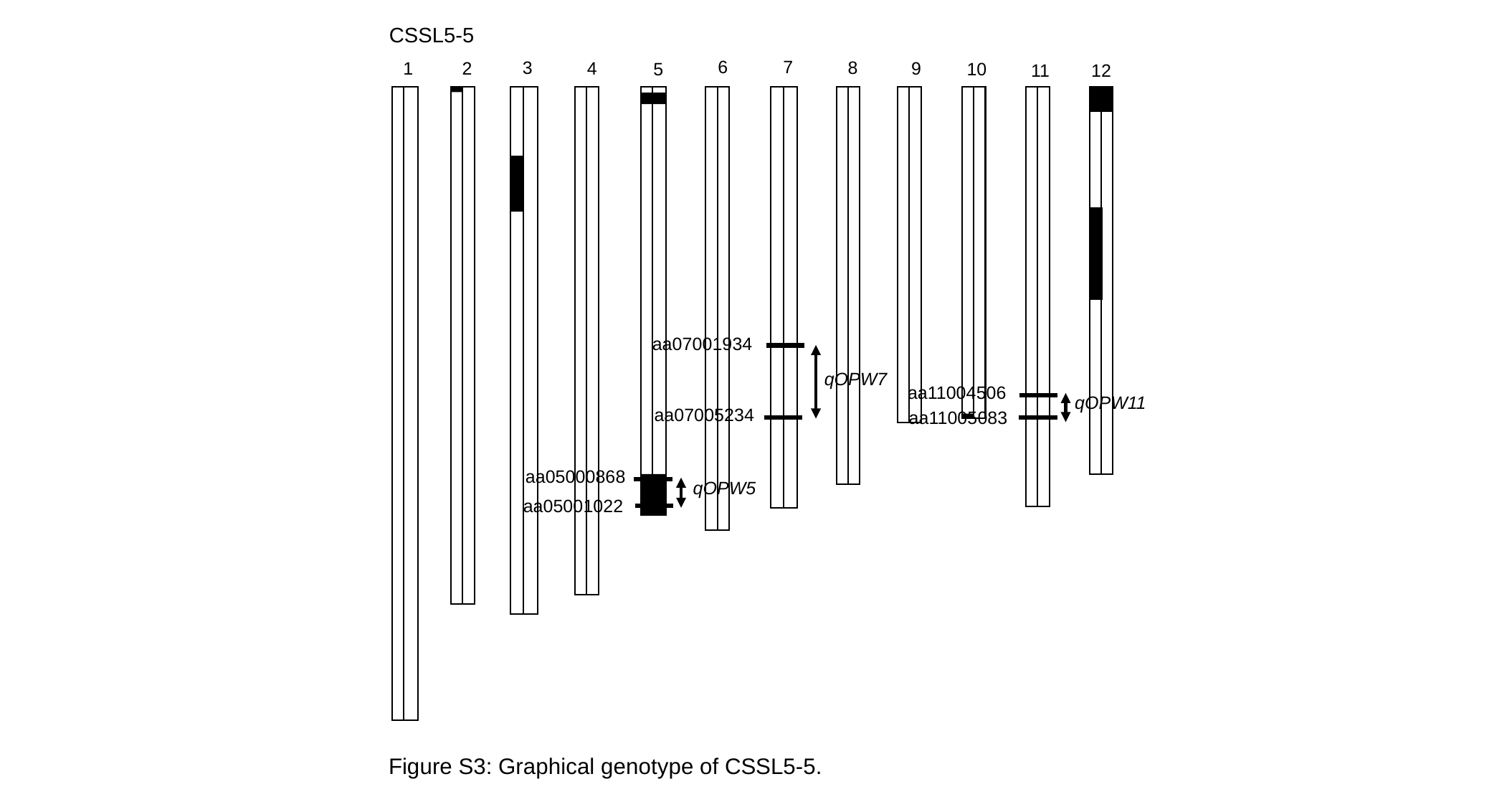

CSSL5-5
6
7
3
8
1
2
4
9
5
10
11
12
aa07001934
qOPW7
aa11004506
qOPW11
aa07005234
aa11005083
aa05000868
qOPW5
aa05001022
Figure S3: Graphical genotype of CSSL5-5.
